# The intersection of Physical Disability and Homelessness: A Community-Engaged Research Initiative

**DOI:** 10.1093/geroni/igaf122.3668

**Published:** 2025-12-31

**Authors:** Natalie Leland, Evan Cole, Max Hurwitz, Julia Lam, Carin Wong, Charity Patterson

**Affiliations:** University of Pittsburgh, Pittsburgh, Pennsylvania, United States; University of Pittsburgh, Pittsburgh, Pennsylvania, United States; UPMC, PIttsburgh, Pennsylvania, United States; Outreach Therapy, Outreach Therapy, Pennsylvania, United States; University of Pittsburgh, University of Pittsburgh, Pennsylvania, United States; University of Pittsburgh, Pittsburgh, Pennsylvania, United States

## Abstract

People experiencing homelessness (PEH), compared to their housed peers, experience higher rates of chronic conditions and accelerated disease progression, which can result in a physical disability (e.g., stroke). This project co-designed and executed an examination of homelessness and healthcare service use and outcomes of PEH with a physical disability compared to their peers. This project was a collaboration between the PEH community and academic researchers, which was guided by a co-created shared governance structure and the 10-Step Framework for Continuous Community Engagement. Collaborative prioritization efforts identified outcomes of interest (e.g., duration of homeless shelter use, 30-day readmissions). To examine homelessness service use among PEH, we leveraged Medicaid claims to identify those who had an index stroke, traumatic brain injury (TBI), or amputation as a surrogate for physical disability and linked to the Homelessness Management Information Systems. To examine healthcare utilization and outcomes we used electronic medical records (EMR) to create a cohort of patients with an index physical disability, stratified by housing status (i.e., PEH vs. housed). The PEH cohort (n = 16,024) included those with an index homeless service event between July 2015 and June 2023, of which 5% (n = 746) had a physical disability. Our one-year EMR cohort included patients experiencing an index physical disability—stroke (n = 9,547), TBI (n = 6,708), or amputation (n = 3,494). PEH had more 30-day readmissions (e.g., 46% vs. 32%, P value=0.01, after TBI) than their housed peers. This collaboration highlights the importance of community partnerships to identify populations to target in future quality initiatives.

